# Trimethoprim-Sulfamethoxazole-Induced Aseptic Meningitis: A New Approach

**DOI:** 10.7759/cureus.15869

**Published:** 2021-06-23

**Authors:** Sarah Elmedani, Asseel Albayati, Ndausung Udongwo, Mihir Odak, Sharif Khawaja

**Affiliations:** 1 Internal Medicine, Jersey Shore University Medical Center, Neptune, USA; 2 Internal Medicine, Jersey Shore University Medical Center, Neptune City, USA

**Keywords:** aseptic meningitis, bactrim, tmp-smx, headache, bacterial meningitis

## Abstract

Inflammation of the meningeal linings of the central nervous system (CNS), also known as meningitis, is one of the serious presentations in the emergency because it carries high morbidity and mortality. The most common cause is pus-producing organisms. However, non-suppurative meningitis, termed aseptic meningitis, is another cause of meningeal inflammation. Many etiologies stand behind aseptic meningitis. Those etiologies include viral and non-viral, drug-induced, malignancy, and systemic inflammation. Drug-induced aseptic meningitis is a rare type of meningitis. Although it is easily treated, it can be a challenging disease if not present in the differential diagnosis. It is commonly associated with nonsteroidal anti-inflammatory drugs (NSAIDs). Nonetheless, other medications have been also reported to cause aseptic meningitis, including antibiotics. Trimethoprim-sulfamethoxazole (TMP-SMX) is one of the most prescribed antibiotics as a prophylactic and therapeutic drug due to its effectiveness and low cost. Although immunocompromised patients are at a higher risk to develop aseptic meningitis, immunocompetent patients are also at risk. Unrelated to the source of the infection, TMP-SMX carries a risk of aseptic meningitis and should be considered as an etiology in patients presenting with meningeal signs and symptoms. Hereby, we report a young immunocompetent patient who developed aseptic meningitis eight days after being prescribed TMP-SMX. Like all drug-induced aseptic meningitis, all his symptoms resolved two days after stopping the medication.

## Introduction

Inflammation of the meninges is most commonly caused by an infectious insult viral meningitis, particularly coxsackieviruses, being the most common type of infectious meningitis [1]. However, noninfectious etiologies are not uncommon autoimmune diseases and medication-induced meningitis has been reported previously in the literature and is termed aseptic meningitis. Drug-induced aseptic meningitis (DIAM) is considered a rare disease, therefore patients who present with atypical meningeal symptoms should have a careful review of the history, examination, and workup. To date, the incidence of DIAM is unknown [2]. It was first described by Weidener and Littman in 1978 in a young woman with SLE who was taking nonsteroidal anti-inflammatory drugs (NSAIDs) [2]. Over the years, several medications other than NSAIDs have been reported to be associated with DIAM, including antibiotics (metronidazole, penicillin, ciprofloxacin, and trimethoprim-sulfamethoxazole (TMP-SMX)) and immunomodulating agents [3]. Trimethoprim-sulfamethoxazole, which is the most commonly reported antibiotic causing DIAM, is a commonly used antibiotic as a prophylactic or definitive treatment of various infectious diseases. Some of its side effects include aplastic anemia, mouth sores, Steven-Johnson syndrome, and aseptic meningitis especially in immunocompromised patients [3]. We report a case of an immunocompetent male without a significant past medical history who presents with signs and symptoms of meningitis after starting TMP-SMX for left leg cellulitis.

## Case presentation

A 51-year-old male presented to the emergency department complaining of headache, subjective fever, and chills for three days. He also reported generalized weakness, diaphoresis, and neck rigidity. He denied cough, shortness of breath, abdominal pain, nausea, vomiting, diarrhea, photophobia, or focal weakness. He had a past medical history of controlled hypertension. Eight days before presentation, he was started on TMP-SMX 800-160 mg tablet two times daily for 10 days for the left foot and ankle redness treated as simple cellulitis.

Vitals were generally stable with a blood pressure of 134/80 mmHg, heart rate of 98 beats per minute, temperature of 97.9℉, respiratory rate of 22 breaths per minute, and oxygen saturation of 99% in room air. Physical examination was remarkable of an uncomfortable, ill-looking patient, with nuchal rigidity, but he was alert and oriented with otherwise unremarkable neurological, lung, and gastrointestinal examination.

The laboratory results are shown in Table 1.

**Table 1:** Laboratory results

Blood	Results	Reference range
WBC	10.8	(4.5-11 x 10^3^/L)
Glucose	116	(70-110 mg/dL)
Blood cultures	Negative	
Procalcitonin (ng/mL)	0.54	(<0.5)
C-reactive protein (mg/dL)	17.54	(0.00 – 0.74 mg/dL)
Albumin (g/dL)	4.0	(3.5 - 5.0 g/dL)
Total bilirubin (mg/dL)	0.5	(0.0 - 1.2 mg/dL)
Aspartate aminotransferase (U/L)	81	(10 - 42 U/L)
Alanine aminotransferase	67	(10 - 60 U/L)
CSF		
Color	colorless	
RBC (/uL)	0	
WBC (/uL)	1	(0 - 5 /uL)
Protein (mg/dL)	54	(14 - 45 mg/dL)
Glucose (mg/dL)	74	(50 - 75 mg/dL)

The non-contrast computed tomography (CT) scan of the head, CT-angiography scan of the chest, and chest X-ray were unremarkable. Blood cultures and urine drug screen were all negative. The coronavirus disease 2019 (COVID-19) test was negative. The right upper quadrant ultrasound and hepatitis panel were unremarkable. Eight hours later, he had a fever of 102℉ and urinalysis was positive showing trace leukocytes. He was initially started on vancomycin and ceftriaxone empirically. Infectious disease (ID) was consulted. Extensive testing for different infectious diseases, including test results for Lyme, malaria, babesia, Ehrlichia chaffeensis, Anaplasma, cytomegalovirus (CMV), HIV, and heterophile antibody, all came back negative. Rheumatoid factor and cyclic citrullinated peptide antibody were all within normal limits.

Also, the meningitis/encephalitis film array result (6 bacteria, 7 viruses, and 1 fungus) was unremarkable. Enzyme-linked immunosorbent assay (ELISA) tests for herpes simplex 1/2 and west Nile viruses were negative. CSF angiotensin-converting enzyme was negative. CSF culture was also negative for bacterial growth. After taking the history and laboratory test results, there was a high index of suspicion for medication-induced aseptic meningitis. TMP-SMX and all other antibiotics were discontinued, and he was monitored and managed conservatively. Two days later, all his symptoms completely resolved, and his aspartate aminotransferase (AST)/alanine transaminase (ALT) levels have improved. He was discharged after five days with instructions to avoid any sulfa medications including TMP-SMX. On his two weeks' outpatient follow-up, the patient remained medically stable and liver enzymes were back to normal.

## Discussion

TMP-SMX is a commonly used antibiotic for several infections involving various systems in the body, including the integumentary system. Partly due to its low cost and bioavailability, many healthcare providers are fond of prescribing this medication to aid in treatment or prophylactic coverage of several bacterial infections. This may be the reason why it has been reported to be the most common antibiotic-induced aseptic meningitis. Aseptic meningitis is one of its rare complications [3]. There are several other medications (DIAM) already reported in the medical literature with this similar complication: NSAIDs, metronidazole, infliximab, lamotrigine, and intravenous immunoglobulins. Other causes of aseptic meningitis include malignancies (leukemias), viruses (CMV), autoimmune diseases (Bechet), and tick-borne borne diseases (Lyme) [4-5]. Therefore, our patient underwent a full workup of these diseases, which all came back negative. This makes DIAM a diagnosis of exclusion, which requires a complete withdrawal (medication challenge test) of the offending drug, just like in our case.

In 1973, the Food and Drug Administration (FDA) approved the use of TMP-SMX, and since its approval, there have been more than 40 cases reported of aseptic meningitis induced by this drug [6-7], mostly reported occurring in women and in patients with autoimmune disorders. There have been few reports of patients taking TMP-SMX for cellulitis. Our patient is a man with no history of autoimmune disorder. TMP and SMX are both weakly bactericidal, by binding to dihydrofolate reductase and dihydropteroate synthetase, respectively. The combined effect of both medications gives a stronger bactericidal effect, thereby disrupting the folic acid pathway that is required for protein synthesis. Its lipophilic nature and low molecular weight enable it to cross the blood-brain barrier, making it a very useful drug in the management of certain CNS infecting bacteria (Toxoplasma gondii, Nocardia, and Listeria) [8]. Immunosuppressed patients (HIV, malignancy, or autoimmune diseases) are at higher risk for the adverse effect of this medication.

The pathophysiology of this drug-inducing meningitis has been proposed by several studies to include either a direct chemical irritation of this drug or hypersensitivity reactions (type 3 or type 4) to the meninges. An immune complex reaction to this medication causing small vessel vasculitis or delayed T-cell activation in the meninges will cause meningeal irritations [9]. Also, since TMP-SMX crosses the blood-brain barrier, the inflammatory response can occur in the central nervous system (meninges), showing meningeal signs that are limited when compared to patients with toxic bacterial meningitis. Diagnosis could be either controlled exposure of the medication in a clinical setting while waiting for symptoms or a withdrawal test to monitor for the resolution of symptoms with the former not being approved, likely due to fear of a devastating outcome. We hope with this case report, we add to the literature more data that signify this drug's side effects. Hopefully, in the near future, new guidelines of criteria can help with early diagnosis of aseptic meningitis without the need to subject the patient to a battery of expensive and invasive investigation, not to mention putting the patient at the risk of developing side effects of other antibacterials [10].

## Conclusions

Early identification of TMP-SMX-induced aseptic meningitis may reduce mortality. A high index of suspicion of this disease is required while taking the past medical history, especially medication history, in patients presenting with fever, headache, and other signs/symptoms of meningitis. We suggest a complete halt in taking this drug if early symptoms of meningitis occur. Symptoms usually resolve within two to three days of discontinuation. To our knowledge, this is the first reported case of TMP-SMX induced aseptic meningitis after cellulitis. This signifies that no matter where the source of infection is, aseptic meningitis should be suspected in patients taking TMP-SMX. We recommend that patients with this disease should avoid all sulfa medications in the future.
